# GDF6 Alleviates Pathological Cardiac Hypertrophy via AMPKα Signaling Pathway

**DOI:** 10.3390/biomedicines13122935

**Published:** 2025-11-29

**Authors:** Quan Ren, Zhiwei Wang, Wei Ren

**Affiliations:** Department of Cardiovascular Surgery, Renmin Hospital of Wuhan University, Jiefang Road 238, Wuhan 430060, China; renquanyymc@163.com (Q.R.); hoslidy@whu.edu.cn (W.R.)

**Keywords:** cardiac hypertrophy, pressure overload, AMPKα, GDF6

## Abstract

**Objective:** Cardiac hypertrophy, a key feature and predisposing factor of heart failure, is mainly controlled by complex signaling cascades. Growth differentiation factor 6 (GDF6) plays critical roles in cell growth and cardiovascular homeostasis; however, its role and underlying mechanisms in cardiac hypertrophy remain unclear. **Methods:** Mice were intravenously injected with adeno-associated virus serotype 9 to overexpress and knock down GDF6 in murine hearts and then exposed to transverse aortic constriction (TAC) surgery to generate pressure overload-induced cardiac hypertrophy. Echocardiographic, histological, and molecular analyses were performed to decipher the alterations to cardiac hypertrophy. In addition, neonatal rat ventricular myocytes (NRVMs) were isolated and stimulated with phenylephrine (PE) to further validate its involvement in hypertrophic growth of cardiomyocytes. **Results:** GDF6 expression was elevated in murine hearts and NRVMs by ROS production under hypertrophic stimuli. GDF6 knockdown aggravated, while GDF6 overexpression attenuated, pressure overload-induced cardiac hypertrophy, inflammation, and dysfunction in vivo. Meanwhile, we found that GDF6 also prevented PE-induced hypertrophic growth of NRVMs in vitro. Mechanistically, GDF6 activated AMPKα to exert cardioprotective effects, and AMPKα inhibition significantly blocked the anti-hypertrophic effects of GDF6. Further studies showed that GDF6 activated AMPKα through the cAMP/Epac1 pathway, and that Epac1 knockdown abolished the protective effects of GDF6 against TAC- or PE-induced cardiac hypertrophy in vivo and in vitro. **Conclusions:** In general, our findings, for the first time, define GDF6 as a negative regulator of cardiac hypertrophy and show that supplementation of GDF6 may be of great therapeutic interest for heart failure.

## 1. Introduction

Cardiac hypertrophy is a key feature and predisposing factor of heart failure, which is characterized by myocyte enlargement, re-activation of fetal gene programs, chronic inflammation, and cardiac dysfunction [1,2,3,4]. Despite extensive studies, the mechanisms of cardiac hypertrophy remain poorly understood. Generally, cardiac hypertrophy is mainly controlled by complex signaling cascades, such as phosphoinositide 3-kinase/protein kinase B (PI3K/AKT), mitogen-activated protein kinases (MAPKs), calcineurin/nuclear factor of activated T cells (CaN/NFATs), etc. [1,5,6,7,8,9]. 5′-adenosine monophosphate-activated protein kinase α (AMPKα) was initially identified as an energy sensor to regulate intracellular metabolic homeostasis; however, emerging studies have defined it as an attractive molecular target to treat various cardiovascular diseases, including cardiac hypertrophy [10,11,12]. Stuck et al. found that angiotensin II (Ang II)-induced cardiomyocyte hypertrophy was accompanied by decreased activation of AMPKα, and that AMPKα activation significantly inhibited cardiomyocyte hypertrophy [13]. Zhang et al. reported that AMPKα deficiency had no effect on cardiac structure or function at baseline, but dramatically increased pressure overload-induced cardiac hypertrophy and dysfunction in mice [14]. Moreover, activating AMPKα also reduced inflammation in hypertrophic hearts and thereby prevented pressure overload-induced cardiac hypertrophy [15,16]. Therefore, activating AMPKα is of great therapeutic interest for treating cardiac hypertrophy and dysfunction.

Growth differentiation factors (GDFs) belong to the evolutionarily conserved transforming growth factor-β superfamily, and are mainly implicated in embryonic development [17]. GDF6 is a member of the GDFs family and plays critical roles in cell growth and cardiovascular homeostasis. Asai-Coakwell et al. detected an increased retinal apoptosis in GDF6-deficient zebrafish embryos [18]. Harrison et al. demonstrated that fibroblast-derived GDF6 facilitated vascular smooth muscle cell growth, thereby aggravating Ang II-induced vascular remodeling and hypertension [19]. In addition, GDF6 overexpression could promote the differentiation of C3H10T1/2 murine embryonic fibroblast cells to cardiomyocyte-like cells in vitro [20]. Wu et al. also found that GDF6 significantly augmented the endothelial angiogenesis and promoted the cardiac function recovery of infarcted hearts [21]. Yet, its role and underlying mechanisms in cardiac hypertrophy remain unclear. The present study tries to overexpress and knock down GDF6 in murine hearts using adeno-associated virus serotype 9 (AAV9) and then establishes transverse aortic constriction (TAC)-induced cardiac hypertrophy to decipher its role in pressure overload-induced cardiac hypertrophy and dysfunction.

In our study, we detect an increased expression of GDF6 in hypertrophic hearts and cardiomyocytes. Gain- and loss-of-function studies indicate that GDF6 knockdown aggravates, while GDF6 overexpression attenuates, pressure overload-induced cardiac hypertrophy, inflammation, and dysfunction in vivo and in vitro. Mechanistically, GDF6 activates AMPKα through cyclic AMP/exchange protein that is directly activated by the cAMP 1 (cAMP/Epac1) pathway to exert cardioprotective effects, and AMPKα inhibition significantly blocks the anti-hypertrophic effects of GDF6. In summary, our findings, for the first time, define GDF6 as a negative regulator of cardiac hypertrophy and show that supplementation of GDF6 may be of great therapeutic interest for heart failure.

## 2. Materials and Methods

### 2.1. Reagents

Nanoparticle In Vivo Transfection Reagent (#5031) was purchased from Altogen Biosystems (Las Vegas, NV, USA). *N*-Acetyl-L-cysteine (NAC, #A7250), apocynin (APO, #178385), compound C (CpC, #171260), 5-bromo-2′-deoxyuridine (BrdU, #B5002), (R)-(-)-phenylephrine hydrochloride (PE, #P6126), 2′,5′-dideoxyadenosine (2′5′-dd-Ado, an adenylyl cyclase/AC inhibitor, #D7408), and H-89 dihydrochloride hydrate (H89, a protein kinase A/PKA inhibitor, #B1427) were purchased from Sigma-Aldrich (St. Louis, MO, USA). Pierce BCA Protein Assay Kit (#23227), TRIzol reagent (#15596026), Lipofectamine RNAiMAX Reagent (#13778150), Goat anti-Rabbit IgG (H + L) Cross-Adsorbed Secondary Antibody, Alexa Fluor™ 568 (#A-11011), and SlowFade™ Gold Antifade Mountant with DAPI (#S36939) were purchased from Invitrogen (Carlsbad, CA, USA). First Strand cDNA Synthesis Kit (#11483188001) and FastStart Universal SYBR Green Master (#4913914001) were purchased from Roche (Basel, Switzerland). Mouse GDF6 ELISA Kit (#MBS034852) was purchased from MyBioSource (San Diego, CA, USA), and recombinant human GDF6 (rhGDF6, #120-04) was purchased from PeproTech (Cranbury, NJ, USA). Mouse IL-1 beta ELISA Kit (#ab197742), Rat IL-1 beta ELISA Kit (#ab255730), Mouse IL-6 ELISA Kit (#ab222503), Rat IL-6 ELISA Kit (#ab234570), Mouse TNF alpha ELISA Kit (#ab208348), Rat TNF alpha ELISA Kit (#ab236712), cAMP Assay Kit (#ab65355), and Protein Kinase A Kinase Activity Assay Kit (#ab139435) were purchased from Abcam (Cambridge, UK). Active Rap1 Detection Kit (#8818) was purchased from Cell Signaling Technology (Danvers, MA, USA). AAV9 carrying short hairpin RNA against GDF6 (AAV9-sh*Gdf6*), full-length mouse GDF6 (AAV9-*Gdf6*), or scramble controls (AAV9-sh*Ctrl* for AAV9-sh*Gdf6* and AAV9-*Ctrl* for AAV9-*Gdf6*) under a cardiomyocyte-specific cTnT promoter were generated by Shanghai GeneChem Co., Ltd. (Shanghai, China). Small interfering RNA against rat Epac1 (si*Epac1*, #SR506839), rat GDF6 (si*Gdf6*, #SR513148), mouse Epac1 (#SR420934), and rat AMPKα2 (si*Ampka2*, #SR508585) were purchased from OriGene (Rockville, MD, USA).

### 2.2. Animals and Experiments

Male C57BL/6 mice (8–12 weeks, 22–28 g) were purchased from Beijing HFK Bioscience Co., Ltd. (Beijing, China) and kept in a SPF environment with controlled temperature (20–25 °C) and humidity (52–58%) under 12/12 h dark/light cycles. All animal experiments were approved by the Animal Welfare & Ethics Committee of our hospital and were also in accordance with the Care and Use of Laboratory Animals (National Institutes of Health Publication No. 85-23, revised 1996). TAC surgery was performed to establish pressure overload-induced cardiac hypertrophy as previously described [2]. Briefly, mice were anesthetized with 2% isoflurane and then exposed to thoracotomy. Next, the transverse aortic arch was surgically isolated and ligated with a 27 G needle using a 7-0 thin thread. Then, the needle was removed, and adequate aortic constriction was verified by Doppler echocardiogram. Mice in sham groups received a similar thoracotomy with no ligation of the transverse aortic arch. To scavenge reactive oxygen species (ROS) in the heart, TAC-operated mice were treated with NAC (500 mg/kg/day) or APO (100 mg/kg/day) in the drinking water as previously described [22]. Four weeks before TAC or sham surgery, mice were intravenously injected with AAV9-sh*Gdf6*, AAV9-*Gdf6*, or matched controls (1 × 10^11^ viral genomes per mouse) to specifically knock down or overexpress GDF6 in murine hearts as previously described [23,24]. To inhibit AMPKα, mice were intraperitoneally injected with 20 mg/kg CpC every other day from 2 weeks after TAC surgery [25]. For *Epac1* gene silence in vivo, mice were injected with chemically modified si*Epac1* using a Nanoparticle In Vivo Transfection Reagent 3 times from 2 weeks after TAC surgery, as previously described [26]. Four weeks after TAC or sham surgery, all mice were euthanized by a cervical dislocation method, with the hearts, lungs, and tibias collected to calculate the heart weight to tibia length (HW/TL) and lung weight/tibia length (LW/TL) ratios.

### 2.3. Echocardiography

Cardiac function was measured by a Vevo 2100 ultrasound echocardiography system (VisualSonics, Toronto, ON, Canada) as previously described [27,28,29]. Briefly, mice were anesthetized by 2% isoflurane, and then two-dimensional parasternal long-/short-axis views as well as an M-mode echocardiogram were captured, with the heart rate, left ventricle internal diameter at end-diastole (LVEDd), left ventricle internal diameter at end-systole (LVEDs), fractional shortening (FS), interventricular septal thickness at end-diastole (IVSd), and interventricular septal thickness at end-systole (IVSs) being calculated from at least 5 consecutive cardiac cycles.

### 2.4. Evaluation of Blood Pressure (BP)

BP was measured using a noninvasive CODA tail-cuff system (Kent Scientific, Litchfield, CT, USA) as previously described [30]. Briefly, mice were adapted and fixed in the pre-heating plate, with the tail or head placed in a hole or the nose fixator of the device, respectively. The pressurized tail sleeve was placed on the tail root, and the sensor was positioned near the pressurized tail sleeve. Next, the program was started, and BP values were recorded.

### 2.5. Western Blot

Total proteins were extracted from murine hearts and cells by RIPA lysis buffer containing a protease inhibitor cocktail and phosphatase inhibitors [31,32,33]. After quantification with a Pierce BCA Protein Assay Kit and denaturation, equal amounts of total proteins were loaded, separated, and transferred onto PVDF membranes. Next, the membranes were blocked, incubated at 4 °C overnight with anti-GDF6 (#ab73288; Abcam), anti-β-actin (#ab8226; Abcam), anti-phospho-AMPKα (p-AMPKα, #2535; CST, Danvers, MA, USA), and anti-total-AMPKα (t-AMPKα, #5832; CST) at a dilution of 1:1000, and then incubated with horse radish peroxidase (HRP)-conjugated secondary antibodies at room temperature for 1 h. Afterwards, the protein bands were visualized via enhanced chemiluminescence and analyzed using Image Lab 6.0 software.

### 2.6. Quantitative Real-Time PCR

Total mRNA was extracted from murine hearts and cells by TRIzol reagent, and then converted to cDNA using a First Strand cDNA Synthesis Kit following the manufacturer’s instructions [34,35,36,37]. Quantitative real-time PCR was performed using a FastStart Universal SYBR Green Master under the following conditions: 95 °C for 5 min, 95 °C for 45 s, and 60 °C for 1 min for 45 cycles. Gene expression was normalized to β-actin (encoded by *Actb*) gene expression, and primer sequences were as follows: *Gdf6* 5′-TAGCTTCCTCTGGGATTTGC-3′; 5′-GAGGAGGAGGACGAGGAGAT-3′; *Actb* 5′-CTGTCGAGTCGCGTCCACCC-3′; 5′-ATGCCGGAGCCGTTGTCGAC-3′; *Nppa* 5′-CACAGATCTGATGGATTTCAAGA-3′; 5′-CCTCATCTTCTACCGGCATC-3′; *Nppb* 5′-ATGGATCTCCTGAAGGTGCTGT-3′; 5′-GCAGCTTGAGATATGTGTCACC-3′; *Myh6* 5′-GCCCAGTACCTCCGAAAGTC-3′; 5′-GCCTTAACATACTCCTCCTTGTC-3′; *Myh7* 5′-CGCATCAAGGAGCTCACC-3′; 5′-CTGCAGCCGCAGTAGGTT-3′; *Epac1* 5′-TCTTACCAGCTAGTGTTCGAGC-3′; 5′-AATGCCGATATAGTCGCAGATG-3′.

### 2.7. GDF6 ELISA Assay

Heart homogenates and cell medium were prepared following the manufacturer’s instructions, added to the 96-well plates, and then incubated with 100 μL HRP-Conjugate Reagent at 37 °C for 1 h. After washing 4 times, 50 µL Chromogen Solution A and 50 µL Chromogen Solution B per well were added to the plates, which were then incubated in the dark at 37 °C for 15 min. This was followed by a reaction with 50 µL Stop Solution. Finally, the optical density was detected at 450 nm by a microplate reader.

### 2.8. Hematoxylin–Eosin Staining

Cross sectional area (CSA) of cardiomyocytes was evaluated by hematoxylin–eosin staining as previously described [38,39,40]. Briefly, hearts were harvested, fixed, dehydrated, embedded, and sectioned into 5 μm thick slices, which were then incubated with hematoxylin or eosin solution following standard protocols. CSA was calculated from more than 100 cells per group using Image-Pro Plus 6.0 software (Media Cybernetics, Bethesda, MD, USA) in a blinded manner.

### 2.9. Cell Isolation and Treatments

Neonatal rat ventricular myocytes (NRVMs) were isolated from 1- to 2-day-old Sprague–Dawley rats and incubated in DMEM/F12 containing 15% fetal bovine serum (FBS) as previously described, and BrdU (0.1 mmol/L) was used to inhibit the proliferation of cardiac fibroblasts in NRVMs [24,41]. To induce hypertrophic growth, NRVMs were incubated with 50 μmol/L PE for 24 h, while phosphate buffer saline (PBS) was used as the negative control. To inhibit ROS in vitro, NRVMs with PE stimulation were treated with NAC (10 mmol/L) or APO (100 µmol/L) as previously described [22]. For GDF6 silence in vitro, NRVMs were transfected with si*Gdf6* (50 nmol/L) for 6 h using a Lipofectamine RNAiMAX reagent, and then cultured in fresh DMEM/F12 containing 15% FBS for an additional 24 h before PE stimulation. Meanwhile, NRVMs were treated with rhGDF6 (4 µg/mL) or dimethyl sulfoxide (DMSO) for 24 h in combination with PE stimulation to further investigate the role of GDF6 in vitro [19]. For AMPKα inhibition, NRVMs were pretreated with 20 μmol/L CpC for 12 h before rhGDF6 treatment [42]. To knock down Epac1, NRVMs were transfected with si*Epac1* (50 nmol/L) for 6 h and cultured for an additional 24 h before rhGDF6 treatment. To inhibit AC or PKA, NRVMs were incubated with 2′5′-ddAdo (200 μmol/L) or H89 (10 μmol/L) in combination with rhGDF6 for 24 h.

### 2.10. Immunofluorescence Staining

Cell area was evaluated by immunofluorescence staining as previously described [43,44,45]. Briefly, NRVMs in coverslips were fixed with 4% formaldehyde for 15 min, permeabilized with 0.2% Triton X-100 for 20 min, blocked with 10% goat serum for 1 h at room temperature, and then incubated with anti-sarcomeric α-actinin (#ab68167, Abcam) at 4 °C overnight. On the second day, coverslips were incubated with Goat anti-Rabbit IgG (H + L) Cross-Adsorbed Secondary Antibody, Alexa Fluor™ 568, and SlowFade™ Gold Antifade Mountant with DAPI for nuclei visualization.

### 2.11. Analysis of Intracellular cAMP Level, PKA Activity, Epac1 Activity and Inflammatory Cytokines

The levels of intracellular cAMP and PKA activity in NRVMs were analyzed using commercial kits, following the manufacturer’s instructions as previously described [46]. To evaluate Epac1 activity, the activity of downstream Rap1 was measured in NRVMs with GDF6 knockdown or supplementation using the Active Rap1 Detection Kit according to the manufacturer’s instructions. The levels of inflammatory cytokines in the myocardium were measured using ELISA kits according to the manufacturer’s instructions.

### 2.12. Statistical Analysis

Results are presented as the mean ± S.D. and analyzed with SPSS 22.0 software. For animal experiments, n indicates different mice. For cell experiments, n indicates independent experiments. Student’s two-tailed *t*-test was performed to compare the means of two-group samples, and one-way analysis of variance (ANOVA) followed by Tukey post hoc test was applied for comparison of multiple groups. *p* < 0.05 was considered significant.

## 3. Results

### 3.1. GDF6 Is Upregulated by ROS Production During Cardiac Hypertrophy

**Figure 1:**
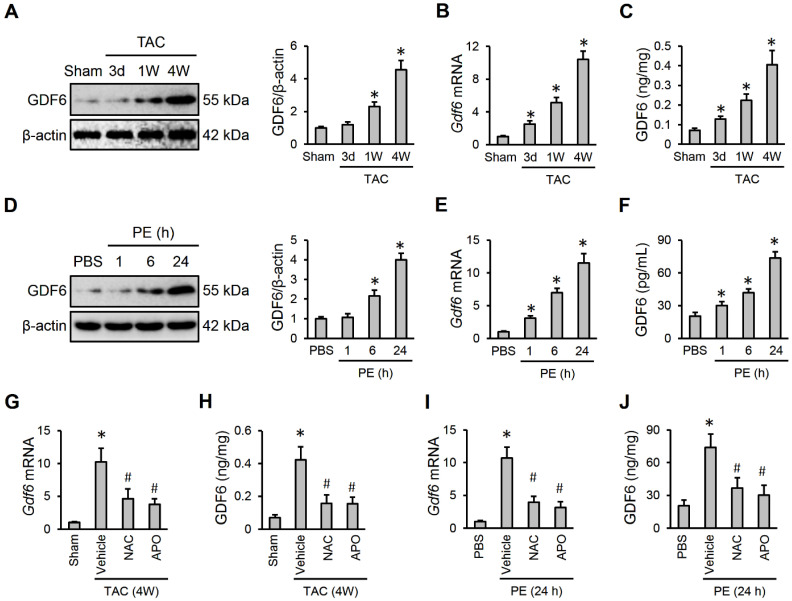
Growth differentiation factor 6 (GDF6) is upregulated by reactive oxygen species (ROS) production during cardiac hypertrophy. (**A**,**B**) GDF6 mRNA and protein levels in murine hearts receiving sham or transverse aortic constriction (TAC) surgery. (**C**) GDF6 levels in murine hearts receiving sham or TAC surgery as determined by an ELISA kit. (**D**,**E**) GDF6 mRNA and protein levels in neonatal rat ventricular myocytes (NRVMs) receiving phosphate buffer saline (PBS) or phenylephrine (PE) stimulation. (**F**) GDF6 levels in the medium from NRVMs receiving PBS or PE stimulation as determined by an ELISA kit. (**G**) GDF6 mRNA levels in murine hypertrophic hearts receiving N-acetyl-L-cysteine (NAC) or apocynin (APO) treatment. (**H**) GDF6 levels in murine hypertrophic hearts receiving NAC or APO treatment as determined by an ELISA kit. (**I**) GDF6 mRNA levels in PE-stimulated NRVMs receiving NAC or APO treatment. (**J**) GDF6 levels in the medium from PE-stimulated NRVMs receiving NAC or APO treatment as determined by an ELISA kit. n = 6 per group. * *p* < 0.05 versus matched sham or PBS groups. In (**G**–**J**), * *p* < 0.05 versus matched sham or PBS groups, # *p* < 0.05 versus matched TAC-operated mice or PE-stimulated NRVMs receiving vehicle treatment. One-way analysis of variance (ANOVA) followed by Tukey post hoc test was applied.

To explore the involvement of GDF6 in the pathogenesis of cardiac hypertrophy, we first determined whether GDF6 expression was altered during cardiac hypertrophy. As shown in Figure 1A,B, GDF6 mRNA and protein levels were gradually elevated in hypertrophic murine hearts. Using an ELISA kit, we also detected an upregulated GDF6 in murine hearts after TAC surgery (Figure 1C). To further clarify the alteration of GDF6, we also detected its expression during PE-induced hypertrophic growth of NRVMs. As shown in Figure 1D,E, GDF6 mRNA and protein levels in NRVMs were significantly elevated by PE stimulation. And the release of GDF6 to medium was also increased in the presence of PE (Figure 1F). ROS is implicated in various cardiovascular diseases, and is significantly elevated in hypertrophic hearts. Therefore, we detected whether ROS production contributed to increased GDF6 expression during cardiac hypertrophy by using the NADPH oxidase inhibitor APO or the ROS scavenger NAC. As shown in Figure 1G–J, both NAC and APO treatment significantly reversed GDF6 upregulation in hypertrophic hearts or NRVMs. These findings indicate that GDF6 is upregulated by ROS production during cardiac hypertrophy.

### 3.2. GDF6 Knockdown Aggravates Pressure Overload-Induced Cardiac Hypertrophy, Inflammation and Dysfunction

**Figure 2:**
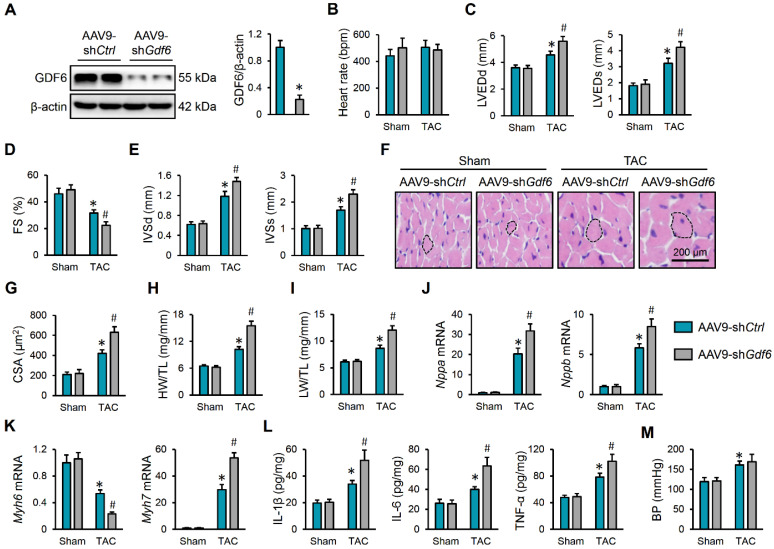
GDF6 knockdown aggravates pressure overload-induced cardiac hypertrophy and dysfunction. (**A**) GDF6 protein levels in murine hearts with AAV9-sh*Ctrl* or AAV9-sh*Gdf6* injection. (**B**) Heart rate in sham- or TAC-operated mice with AAV9-sh*Ctrl* or AAV9-sh*Gdf6* injection. (**C**,**D**) Left ventricle internal diameter at end-diastole (LVEDd), left ventricle internal diameter at end-systole (LVEDs), and fractional shortening (FS). (**E**) Interventricular septal thickness at end-diastole (IVSd) and interventricular septal thickness at end-systole (IVSs). (**F**,**G**) Hematoxylin–eosin staining of murine hearts and quantification of cross-sectional area (CSA). (**H**,**I**) Quantification of heart weight/tibia length (HW/TL) and lung weight/tibia length (LW/TL) ratios. (**J**,**K**) *Nppa*, *Nppb*, *Myh7*, and *Myh6* mRNA levels in murine hearts. (**L**) The levels of inflammatory cytokines in the myocardium. (**M**) BP levels. n = 6 per group. * *p* < 0.05 versus matched sham mice receiving AAV9-sh*Ctrl* injection, # *p* < 0.05 versus matched TAC mice receiving AAV9-sh*Ctrl* injection. One-way ANOVA followed by Tukey post hoc test was applied. In (**A**), Student’s two-tailed *t*-test was performed.

Next, mice were intravenously injected with AAV9-sh*Gdf6* to specifically knock down GDF6 in murine hearts, and the efficiency was verified by Western blot (Figure 2A). As shown in Figure 2B–D, GDF6 knockdown did not affect the mice’s heart rate but further aggravated TAC-induced cardiac dilation and dysfunction in mice, as evidenced by increased LVEDd, LVEDs, and FS. Echocardiographic analysis also determined significant elevations of IVSd and IVSs in GDF6-silenced hearts, indicating exacerbated cardiac hypertrophy (Figure 2E). Consistently, hematoxylin–eosin staining revealed that GDF6 knockdown accelerated the hypertrophic growth of cardiomyocytes in response to pressure overload (Figure 2F,G). Meanwhile, we also detected increases in the HW/TL and LW/TL ratios in mice with AAV9-sh*Gdf6* injection, which indicates an aggravated cardiac hypertrophy and dysfunction (Figure 2H,I). In line with the phenotypic alterations, we found that GDF6 knockdown significantly elevated the mRNA levels of *Nppa*, *Nppb*, and *Myh7*, but further reduced the *Myh6* mRNA level (Figure 2J,K). Chronic inflammation is a key feature and pathogenic factor of cardiac hypertrophy. Interestingly, we found that the increased levels of IL-1β, IL-6, and TNF-α in TAC-induced hearts were further elevated by GDF6 silence (Figure 2L). A previous study showed that GDF6 could facilitate vascular smooth muscle cell growth and that it aggravated Ang II-induced vascular remodeling and hypertension [19]. However, we found that GDF6 knockdown in murine hearts made no alteration to BP (Figure 2M). Collectively, we demonstrate that GDF6 knockdown aggravates pressure overload-induced cardiac hypertrophy and dysfunction.

### 3.3. GDF6 Overexpression Attenuates Pressure Overload-Induced Cardiac Hypertrophy, Inflammation and Dysfunction

**Figure 3:**
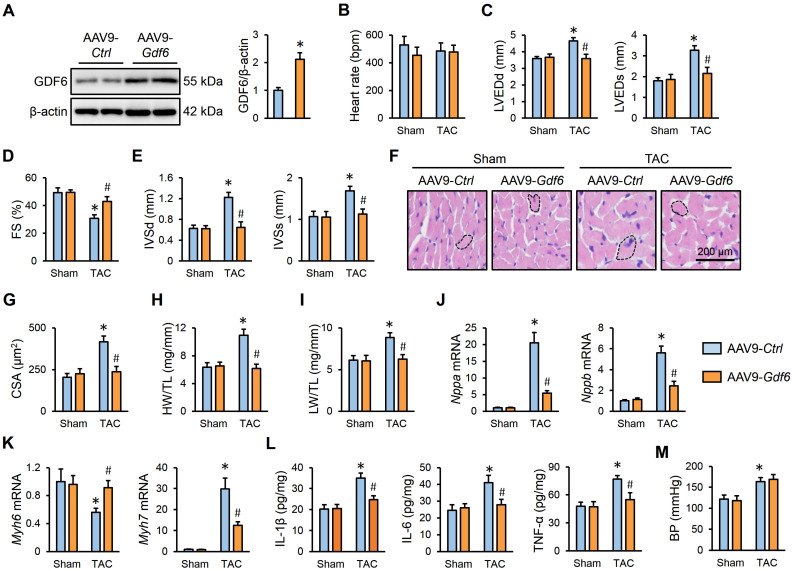
GDF6 overexpression attenuates pressure overload-induced cardiac hypertrophy and dysfunction. (**A**) GDF6 protein levels in murine hearts injected with AAV9-*Ctrl* or AAV9-*Gdf6*. (**B**) Heart rate in sham- or TAC-operated mice with AAV9-*Ctrl* or AAV9-*Gdf6* injection. (**C**,**D**) LVEDd, LVEDs, and FS. (**E**) IVSd and IVSs. (**F**,**G**) Hematoxylin–eosin staining of murine hearts and quantification of CSA. (**H**,**I**) Quantification of HW/TL and LW/TL. (**J**,**K**) *Nppa*, *Nppb*, *Myh7*, and *Myh6* mRNA levels in murine hearts. (**L**) The levels of inflammatory cytokines in the myocardium. (**M**) BP levels. n = 6 per group. * *p* < 0.05 versus matched sham mice receiving AAV9-*Ctrl* injection, # *p* < 0.05 versus matched TAC mice receiving AAV9-*Ctrl* injection. One-way ANOVA followed by Tukey post hoc test was applied. In (**A**), Student’s two-tailed *t*-test was performed.

Then, mice were intravenously injected with AAV9-*Gdf6* to specifically overexpress GDF6 in murine hearts, and the efficiency was verified by Western blot (Figure 3A). As shown in Figure 3B–D, GDF6 overexpression did not affect the heart rate, but significantly attenuated TAC-induced cardiac dilation and dysfunction in mice, as evidenced by decreased LVEDd, LVEDs, and FS. Echocardiographic analysis also determined significant decreases in the IVSd and IVSs in GDF6-overexpressed hearts, which indicates an improved cardiac hypertrophy (Figure 3E). Consistently, hematoxylin–eosin staining revealed that GDF6 overexpression delayed the hypertrophic growth of cardiomyocytes in response to pressure overload (Figure 3F,G). Meanwhile, we also detected decreases in HW/TL and LW/TL in mice with AAV9-*Gdf6* injection, which indicates an improved cardiac hypertrophy and dysfunction (Figure 3H,I). And the dysregulated mRNA expressions of hypertrophic markers were also restored by GDF6 overexpression (Figure 3J,K). Meanwhile, we found that the increased levels of IL-1β, IL-6, and TNF-α in TAC-induced hearts were dramatically inhibited by GDF6 overexpression (Figure 3L). In addition, we found that GDF6 overexpression in murine hearts also made no alteration to BP (Figure 3M). Taken together, our data reveal that GDF6 overexpression attenuates pressure overload-induced cardiac hypertrophy and dysfunction.

### 3.4. GDF6 Prevents PE-Induced Hypertrophic Growth of NRVMs

**Figure 4:**
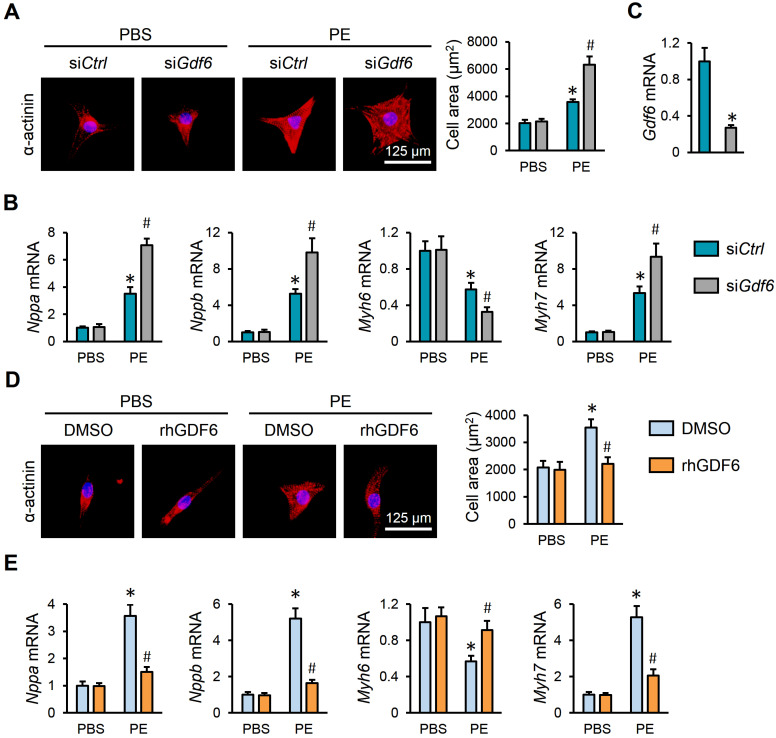
GDF6 prevents PE-induced hypertrophic growth of NRVMs. (**A**) Immunofluorescence staining of α-actinin and quantification of cell area in NRVMs with or without GDF6 knockdown. (**B**) *Nppa*, *Nppb*, *Myh7*, and *Myh6* mRNA levels in NRVMs with or without GDF6 knockdown. (**C**) GDF6 mRNA levels in NRVMs with or without GDF6 knockdown. (**D**) Immunofluorescence staining of α-actinin and quantification of cell area in NRVMs with or without recombinant human GDF6 (rhGDF6) treatment. (**E**) *Nppa*, *Nppb*, *Myh7*, and *Myh6* mRNA levels in NRVMs with or without rhGDF6 treatment. n = 6 per group. * *p* < 0.05 versus matched PBS NRVMs receiving si*Ctrl* or dimethyl sulfoxide (DMSO) treatment, # *p* < 0.05 versus matched PE NRVMs receiving si*Ctrl* or DMSO treatment. One-way ANOVA followed by Tukey post hoc test was applied. In (**C**), Student’s two-tailed *t*-test was performed.

Additionally, NRVMs were isolated and stimulated with PE to further evaluate the role of GDF6 in vitro. As shown in Figure 4A, GDF6 knockdown significantly aggravated PE-induced hypertrophic growth of NRVMs. Meanwhile, the dysregulated mRNA levels of hypertrophic markers were further disarranged by GDF6 knockdown (Figure 4B,C). In contrast, supplementation with rhGDF6 significantly prevented PE-induced hypertrophic growth of NRVMs, as evidenced by a decreased cell area, decreased *Nppa*, *Nppb*, and *Myh7* mRNA levels, and an increased *Myh6* mRNA level (Figure 4D,E). These results suggest that GDF6 prevents PE-induced hypertrophic growth of NRVMs.

### 3.5. GDF6 Ameliorates Cardiac Hypertrophy Through Activating AMPKα In Vivo and In Vitro

**Figure 5:**
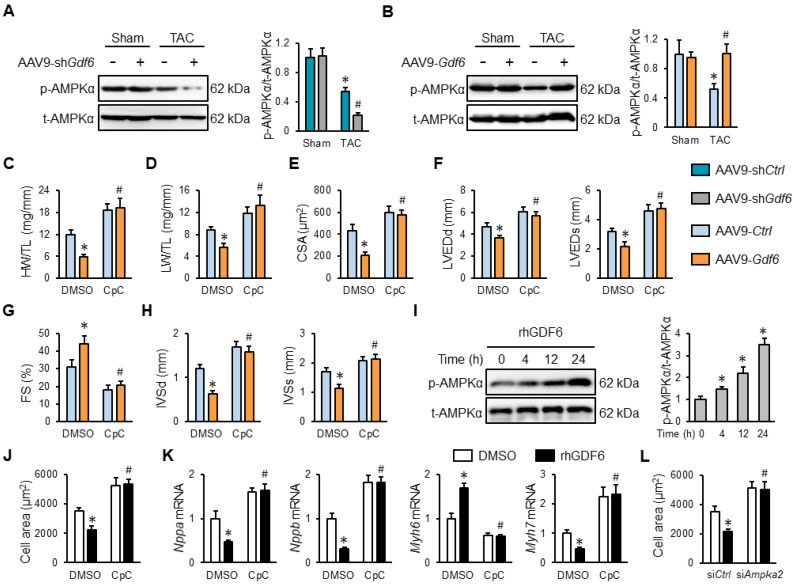
GDF6 ameliorates cardiac hypertrophy through activating 5′-adenosine monophosphate-activated protein kinase α (AMPKα) in vivo and in vitro. (**A**) AMPKα protein levels in sham- or TAC-operated murine hearts with AAV9-sh*Ctrl* or AAV9-sh*Gdf6* injection. (**B**) AMPKα protein levels in sham- or TAC-operated murine hearts with AAV9-*Ctrl* or AAV9-*Gdf6* injection. (**C**,**D**) HW/TL and LW/TL in TAC-operated mice injected with AAV9-sh*Ctrl* or AAV9-sh*Gdf6* in the presence or absence of compound C (CpC). (**E**) Quantification of CSA. (**F**,**G**) FS, LVEDd, and LVEDs. (**H**) IVSd and IVSs. (**I**) AMPKα protein levels in rhGDF6-treated NRVMs at different time points. (**J**) Quantification of cell area in PE-stimulated NRVMs with or without rhGDF6 treatment in the presence or absence of CpC. (**K**) *Nppa*, *Nppb*, *Myh7*, and *Myh6* mRNA levels in NRVMs. (**L**) Quantification of cell area in PE-stimulated NRVMs with or without rhGDF6 treatment in the presence or absence of si*Ampka2* transfection. n = 6 per group. * *p* < 0.05 versus matched AAV9-*Ctrl*-injected TAC mice receiving DMSO treatment, # *p* < 0.05 versus matched AAV9-*Gdf6*-injected TAC mice receiving DMSO treatment. In (**A**,**B**), * *p* < 0.05 versus matched sham mice receiving AAV9-sh*Ctrl* or AAV9-*Ctrl* injection, # *p* < 0.05 versus matched TAC mice receiving AAV9-sh*Ctrl* or AAV9-*Ctrl* injection. In (**I**,**J**), * *p* < 0.05 versus matched DMSO-stimulated NRVMs under PE incubation, # *p* < 0.05 versus matched DMSO-stimulated NRVMs with rhGDF6 treatment under PE incubation. One-way ANOVA followed by Tukey post hoc test was applied.

AMPKα is an attractive molecular target to treat cardiac hypertrophy, and we herein investigated whether it was required for GDF6-mediated protection against cardiac hypertrophy [14]. As shown in Figure 5A,B, GDF6 knockdown decreased, while GDF6 overexpression increased, the AMPKα phosphorylation in hypertrophic hearts. To further validate the necessity of AMPKα, mice in TAC groups were intraperitoneally injected with 20 mg/kg CpC every other day from 2 weeks after TAC surgery to inhibit AMPKα [25]. As shown in Figure 5C,D, the inhibitory effects of GDF6 on the HW/TL and LW/TL ratios in TAC mice were completely blocked by CpC. And GDF6 overexpression also failed to prevent TAC-induced cardiomyocyte hypertrophy in the presence of CpC, as evidenced by increased CSA (Figure 5E). Meanwhile, echocardiographic analysis also revealed that CpC treatment significantly blocked GDF6 overexpression-mediated protective effects against ventricular hypertrophy, dilation, and dysfunction, as evidenced by decreased FS and increased LVEDd, LVEDs, IVSd, and IVSs (Figure 5F–H). In addition, we also found that rhGDF6 activated AMPKα in a time-dependent manner (Figure 5I). Consistent with the in vivo findings, we demonstrated that rhGDF6-mediated inhibitory effects against PE-induced hypertrophic growth of NRVMs were also prevented by CpC treatment in vitro (Figure 5J,K). Moreover, AMPKα knockdown also prevented the inhibitory effects of rhGDF6 in PE-induced cardiomyocyte hypertrophy, as evidenced by the increased cell area (Figure 5L). Our study implies that GDF6 ameliorates cardiac hypertrophy through activating AMPKα in vivo and in vitro.

### 3.6. GDF6 Activates AMPKα Through cAMP/Epac1 Pathway

**Figure 6:**
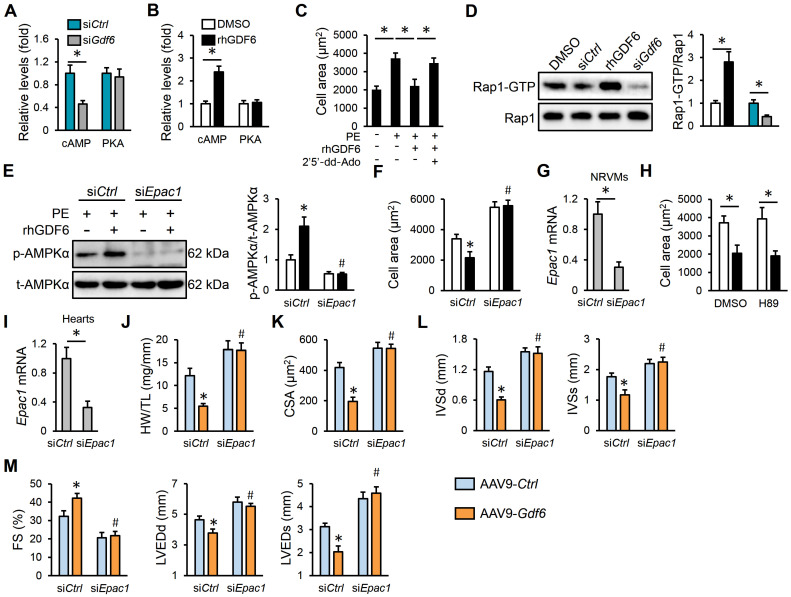
GDF6 activates AMPKα through cyclic AMP/exchange protein directly activated by cAMP 1 (cAMP/Epac1) pathway. (**A**) The levels of cAMP and protein kinase A (PKA) activity in PE-stimulated NRVMs with or without GDF6 knockdown. (**B**) The levels of cAMP and PKA activity in PE-stimulated NRVMs with or without rhGDF6 treatment. (**C**) Quantification of cell area in rhGDF6-treated NRVMs with or without adenylyl cyclase (AC) inhibition. (**D**) Rap1-GTP and total Rap1 protein levels in PE-stimulated NRVMs treated with si*Gdf6* or rhGDF6. (**E**) AMPKα protein levels in PE-stimulated NRVMs with or without Epac1 silence in the presence or absence of rhGDF6. (**F**) Quantification of cell area. (**G**) Epac1 mRNA levels in NRVMs with or without Epac1 silence. (**H**) Quantification of cell area. (**I**) Epac1 mRNA levels in murine hearts with or without Epac1 silence. (**J**) HW/TL in TAC mice with or without Epac1 silence in the presence or absence of AAV9-*Gdf6*. (**K**) Quantification of CSA. (**L**) IVSd and IVSs. (**M**) FS, LVEDd, and LVEDs. n = 6 per group. * *p* < 0.05 versus matched AAV9-*Ctrl*-injected TAC mice receiving si*Ctrl* injection, # *p* < 0.05 versus matched AAV9-*Gdf6*-injected TAC mice receiving si*Ctrl* injection. In (**A**–**D**,**G**–**I**), * *p* < 0.05 versus the matched groups. In (**E**,**F**), * *p* < 0.05 versus matched si*Ctrl*-transfected NRVMs with DMSO treatment under PE incubation, # *p* < 0.05 versus matched si*Ctrl*-transfected NRVMs with rhGDF6 treatment under PE incubation. One-way ANOVA followed by Tukey post hoc test was applied. In (**A**,**B**,**D**,**G**–**I**), Student’s two-tailed *t*-test was performed.

Finally, we explored the precise mechanism through which GDF6 activated AMPKα. The cAMP is a critical secondary messenger in transducing extracellular stimuli to intracellular signaling network, and acts as a classic upstream activator of AMPKα in a PKA-dependent manner [47]. As shown in Figure 6A,B, GDF6 knockdown decreased, while rhGDF6 treatment increased, intracellular cAMP levels in PE-stimulated NRVMs, without affecting PKA activities. However, the protective effects of rhGDF6 against PE-induced cardiomyocyte hypertrophy were prevented by 2′5′-dd-Ado, an AC inhibitor that reduces cAMP generation (Figure 6C). In addition to PKA, Epac1, the other downstream effector of cAMP, is a well-known activator of AMPKα [48,49]. We found that Epac1 was activated by rhGDF6, but inhibited by GDF6 knockdown, as evidenced by the downstream Rap1-GTP levels (Figure 6D). As shown in Figure 6E, Epac1 silence completely abolished rhGDF6-mediated AMPKα activation under PE stimulation. As expected, the anti-hypertrophic effects of rhGDF6 were also blocked in PE-treated NRVMs with Epac1 silence (Figure 6F,G). As expected, PKA inhibition did not affect the anti-hypertrophic effects of rhGDF6 in vitro (Figure 6H). To further validate the role of Epac in vivo, TAC mice were delivered with chemically modified si*Epac1* using a Nanoparticle In Vivo Transfection Reagent from 2 weeks after TAC surgery, and the efficiency was validated by quantitative real-time PCR (Figure 6I). Consistent with the in vitro findings, Epac1 silence also blocked the anti-hypertrophic effects of GDF6 in mice, as evidenced by an increased HW/TL, CSA, IVSd, and IVSs (Figure 6J–L). Meanwhile, the improved cardiac dilation and dysfunction in GDF6-overexpressed hypertrophic hearts were also abolished by Epac1 silence (Figure 6M). Overall, our results reveal that GDF6 activates AMPKα through the cAMP/Epac1 pathway.

## 4. Discussion

Cardiac hypertrophy initially occurs as an adaptive response to maintain normal cardiac function and output under various pathological stimuli, which eventually predisposes to the occurrence of heart failure [1,2]. Generally, hypertension acts as the major contributor of hypertrophic growth of the heart, and TAC-induced pressure overload-associated cardiac hypertrophy is a well-accepted pathological model to investigate the molecular basis of cardiac hypertrophy [2]. In the present study, we established TAC-induced cardiac hypertrophy in mice and PE-induced hypertrophic growth of NRVMs, and detected an elevated GDF6 expression in murine hearts and NRVMs under hypertrophic stimuli. GDF6 knockdown aggravated, while GDF6 overexpression attenuated, pressure overload-induced cardiac hypertrophy and dysfunction in vivo. Meanwhile, we found that GDF6 also prevented PE-induced hypertrophic growth of NRVMs in vitro. Mechanistically, GDF6 activated AMPKα to exert the cardioprotective effects, and AMPKα inhibition significantly blocked the anti-hypertrophic effects of GDF6. Further studies showed that GDF6 activated AMPKα through the cAMP/Epac1 pathway, and that Epac1 knockdown abolished the protective effects of GDF6 against TAC- or PE-induced cardiac hypertrophy in vivo and in vitro. In general, our findings, for the first time, define GDF6 as a negative regulator cardiac hypertrophy and show that supplementation of GDF6 may be of great therapeutic interest for heart failure.

The regulation of cardiac hypertrophy is orchestrated by complex signaling cascades (e.g., PI3K/AKT, MAPKs, and CaN/NFATs), and targeting these pathways provides significant cardioprotections against hypertrophic growth of the heart [1]. Inflammation is also implicated in the pathogenesis of hypertrophic growth of the heart. AMPKα emerges as an attractive and effective molecular node to reduce inflammation and maintain cardiac homeostasis under different pathological stimuli. Zhang et al. previously found that AMPKα activation blocked the transdifferentiation and collagen synthesis of cardiac fibroblasts, leading to an improved fibrotic remodeling and cardiac dysfunction [41]. And Hu et al. recently also demonstrated that AMPKα activation inhibited aging-related inflammation, oxidative stress, and cardiac hypertrophy and dysfunction [48]. In addition, AMPKα activation also prevented cardiac hypertrophy and dysfunction. Li et al. reported that AMPKα activation stimulated autophagy and significantly blocked PE-induced cardiomyocyte hypertrophy [50]. Further, AMPKα activation also reduced protein O-GlcNAcylation and then counteracted cardiac hypertrophy [51]. Moreover, Nam et al. demonstrated that transient AMPKα activation before pressure overload could also effectively prevent adverse remodeling and cardiac dysfunction in mice [52]. In contrast, Myers et al. showed that treatment with a potent, direct, and allosteric activator of AMPKα, MK-8722, significantly improved glucose homeostasis but induced cardiac hypertrophy across species [53]. Therefore, it is of great therapeutic interest to find novel and specific activators of AMPKα for the treatment of cardiac hypertrophy and dysfunction, especially endogenous activators. In this study, we observed a significant upregulation of GDF6 in hypertrophic hearts and NRVMs, and that GDF6 overexpression or supplementation effectively blocked TAC- or PE-induced cardiac hypertrophy in vivo and in vitro. AMPKα acts as a sensor of cellular energy status and is activated by increases in the cellular AMP/ATP ratio under metabolic stresses. Commonly, AMPKα is directly activated by the known upstream kinases, including LKB1 and CaMKKβ. GDF6 is a secretory factor, and transduces extracellular stimuli to the intracellular molecular network through membrane receptors and secondary messengers. cAMP is an important second messenger and upstream activator of AMPKα [47]. Roth et al. demonstrated that restoring the cAMP-generating capacity could improve cardiac hypertrophy and dysfunction in mice [54]. In addition, cardiomyocyte-secreted cAMP could be converted into its metabolite adenosine and then reduce cardiac hypertrophy and fibrosis through adenosine receptors [55]. In contrast, other studies argued that cAMP could facilitate cardiac hypertrophy, and this discrepancy could be attributable to its different source [56,57]. Interestingly, previous studies have shown that the precise role of cAMP depends on where it is made. Different receptors producing spatially segregated pools of cAMP in discrete subcellular locations do not move freely throughout the cell, and subsequently trigger compartmentalized signaling pathways in cardiomyocytes [58,59]. Accordingly, β1-adrenergic receptor (AR) enhanced PKA-dependent phosphorylation of multiple target proteins, including L-type Ca2+ channels (LTCCs) and phospholamban; however, β2AR induces a cAMP-dependent response limited to LTCCs regulation [60]. PKA is a well-known downstream effector of cAMP and contributes to the progression of cardiac hypertrophy [61]. Interestingly, we found no alteration of PKA activity in PE-stimulated NRVMs with either GDF6 knockdown or rhGDF6 treatment. Instead, Epac1, the other target of cAMP, was required for the activation of AMPKα by GDF6. Consistently, Laurent et al. determined that stimulating Epac1 activated AMPKα-dependent autophagy, thereby preventing cardiomyocyte hypertrophy [62]. Herein, we observed that Epac1 silence abolished GDF6-mediated AMPKα activation and anti-hypertrophic effects in vivo and in vitro. Currently, no definite receptors for GDF6 have been identified. GDF6 belongs to the transforming growth factor-β superfamily, and potential receptors for GDFs have been identified, such as glial cell-derived neurotrophic factor receptor alpha-like, bone morphogenetic protein receptors, epidermal growth factor receptor, platelet-derived growth factor receptor, etc. In our study, we found that reducing cAMP generation with the AC inhibitor dramatically abolished the anti-hypertrophic role of rhGDF6, which indicates the potential involvement of G protein-coupled receptors in GDF6. Further studies are needed to identify the specific receptors for GDF6. Based on these findings, we propose that cAMP generated by the specific receptor for GDF6 induces a biased activation of the Epac1/AMPKα pathway.

In our study, neither GDF6 overexpression nor GDF6 knockdown affected cardiac morphology or function at basal conditions, which indicated that GDF6 manipulation did not induce any significant side effects. Meanwhile, treatment with recombinant human GDF6 also did not affect the cell morphology or viability in rat cardiomyocytes. These in vivo and in vitro data identified the safety of GDF6 manipulation for the treatment of cardiac hypertrophy and heart failure. For the possible off-target effects from systemic GDF6 administration, gene therapy with viral or non-viral vectors can be used in future studies. Of note, there are some limitations of the present study. First, the specific molecular mechanisms mediating GDF6 upregulation by ROS remain unclear. Second, whether GDF6 expressed by other cardiac cells also prevents pressure overload-induced cardiac hypertrophy and heart failure should be determined in further studies. Third, the precise receptor for GDF6 and the downstream compartmentalized cAMP signaling pathways in cardiomyocytes are elusive.

In general, our findings, for the first time, define GDF6 as a negative regulator of cardiac hypertrophy and show that supplementation of GDF6 with viral/non-viral vectors or systemic administration with nanoparticles may be of great therapeutic interest for heart failure.

## Data Availability

The datasets used and/or analyzed during this study are available from the corresponding author on reasonable request.
